# A cell atlas of the adult female *Aedes aegypti* midgut revealed by single-cell RNA sequencing

**DOI:** 10.1038/s41597-024-03432-8

**Published:** 2024-06-05

**Authors:** Shunlong Wang, Ying Huang, Fei Wang, Qian Han, Nanjie Ren, Xiaoyu Wang, Yingjun Cui, Zhiming Yuan, Han Xia

**Affiliations:** 1 Key Laboratory of Virology and Biosafety, Wuhan Institute of Virology, Chinese Academy of Sciences, Wuhan, 430200 China; 2 University of Chinese Academy of Sciences, Beijing, 100049 China; 3 Hainan One Health Key Laboratory, Hainan University, Haikou, 570228 China; 4 Section of Infectious Diseases, Department of Internal Medicine, Yale School of Medicine, New Haven, 06520 USA; 5 Hubei Jiangxia Laboratory, Wuhan, 430207 China

**Keywords:** Entomology, Genetic databases, Sequence annotation, Viral vectors

## Abstract

*Aedes aegypti* is a primary vector for transmitting various arboviruses, including Yellow fever, dengue and Zika virus. The mosquito midgut is the principal organ for blood meal digestion, nutrient absorption and the initial site of arbovirus infection. Although a previous study delineated midgut’s transcriptome of *Ae. aegypti* at the single-nucleus resolution, there still lacks an established protocol for isolating and RNA sequencing of single cells of *Ae. aegypti* midgut, which is required for investigating arbovirus-midgut interaction at the single-cell level. Here, we established an atlas of the midgut cells for *Ae. aegypti* by single-cell RNA sequencing. We annotated the cell clusters including intestinal stem cells/enteroblasts (ISC/EB), cardia cells (Cardia), enterocytes (EC, EC-like), enteroendocrine cells (EE), visceral muscle (VM), fat body cells (FBC) and hemocyte cells (HC). This study will provide a foundation for further studies of arbovirus infection in mosquito midgut at the single-cell level.

## Background & Summary

Mosquitoes are hematophagous arthropods transmitting various pathogens^1^. *Aedes aegypti* is a mosquito species that transmits arboviruses, including dengue, Zika and Chikungunya, posing severe threats to public health globally^2,3^. The female mosquito’s midgut is the primary digestive and absorption organ, it is also involved in many physiological processes like oogenesis, signal molecule secretion, gut microbiota shaping and immune reactions^4,5^. Anatomically, the female mosquito’s midgut is spindle-shaped and a single inner layer of epithelial cells on a basal lamina surrounded by visceral muscles, tracheoles and fibroblasts embedded in the outer extracellular matrix^4,5^. Successful arbovirus infection necessitates overcoming two barriers in the midgut: the midgut infection barrier and the midgut escape barrier, both of which influence vector competence^6^. In arbovirus infection, the midgut is the initial infection site and plays a dual role in pathogen transmission and defense against pathogen invasion^7^. The cells from different midgut regions exhibit different proviral or antiviral effects, indicating arboviruses have cell type-specific preference in host-pathogen interactions^8,9^.

Decades of advancements in bulk RNA sequencing technologies (bulk RNA-seq) have significantly contributed to the field of transcriptomics. Studies have shown that transcriptomes may differ among cells or between tissues from the same organ. However, bulk RNA-seq has limitations, as it only provides an overall average of gene expression, thereby hiding unique transcriptional differences at the cellular level^10,11^. In contrast, single-cell RNA sequencing (scRNA-seq) technologies allow the study of transcriptome heterogeneity in a cell-to-cell resolution^12^. The cells with similar function or morphology could be separated based on transcriptional heterogeneity^10,11^. Therefore, scRNA-seq has been widely used to reveal cellular composition, discover novel or rare cell types, and characterize gene expression changes during cell differentiation or other stages. For example, a single-cell study of mosquito hemocytes revealed the diversity of hemocyte functions, identified different cell subtypes such as hemocytes (HC) and fat body cells (FBC) separately, and illuminated the developmental trajectories from granulocytes to antimicrobial granulocytes^13^.

Previous studies on the midgut of *Drosophila*, *Ae. aegypti* and other *Diptera* insects could serve as references for annotating mosquito midgut cells^5,14,15^. The midgut cells of adult dipteran insects consist of four epithelial cell types: enterocytes (EC), enteroendocrine cells (EE), enteroblasts (EB), and intestinal stem cells (ISC), and two other cell types called cardia cells (Cardia) and visceral muscles (VM)^14,15^. Unlike mammalian ECs, insect ECs fulfil roles in both nutrient transport and digestive enzyme secretion^5^. EEs produce and secret bioactive peptides and other signal molecules, playing vital regulatory roles in midgut functionality and insect homeostasis^16^. EBs act as progenitors of ECs and EEs, originating from ISCs via asymmetric division. ISCs can undergo symmetric division to generate either two stem cells or two differentiated cells, thereby maintaining a self-renewing epithelium under homeostatic conditions or in response to stressors in the life cycle^17^. Cardia cells are located at the junction between the foregut and midgut, while VMs facilitate food movement from the midgut to the hindgut^5^. But it should be noted that there is a major difference between *Drosophila* and mosquitoes, as hematophagy has distinct metabolic requirements that will contribute to the transcriptome^14,15,18,19^.

Cellular heterogeneity affects vector competence and host-pathogen interaction. scRNA-seq study of *Anopheles gambiae* hemocytes revealed a novel cell type called “megacyte” that plays a crucial role in response to the invasion of *Plasmodium* parasite^13^. Sindbis virus (SINV) prefers infecting EEs in the midgut of *Ae. aegypti* and may utilize the neurosecretory nature of EEs to disseminate to neighboring midgut cells and other target tissues^9^. Therefore, establishing a cell atlas of *Ae. aegypti* midgut at single-cell level can provide a reference for future studies on arbovirus-midgut interaction.

An earlier study utilizing single-nucleus RNA sequencing technology (snRNA-seq) identified and annotated 20 distinguishable cell clusters including ISC/EB, EE, EC, cardia, and VM from the midgut of *Ae. aegypti*^15^. Analyzing single nucleus rather than single cells is an important strategy that addresses tissues that cannot be readily dissociated into a single-cell suspension and frozen samples^20^. But snRNA-seq cannot detect mRNAs in the cytoplasm. Since some transcripts are not mainly located in the nucleus and the higher proportion of mature mRNA with polyA tails exist in the cytoplasm, the scRNA-seq has great potential in the identification of cell subtypes and the annotation of the pathogen-infected mosquito cells^21,22^. Here, we performed scRNA-seq experiments on mosquito midguts and successfully identified the major cell types. Our study will aid in advancing further scRNA-seq studies of the arbovirus-infected mosquito midgut.

## Methods

### Mosquitoes

The eggs of *Ae. aegypti* (Rockefeller strain) were hatched in a transparent box containing distilled water, and the larvae were fed with fish feed (Bessn, China) at 28 °C with 12 h:12 h light/dark cycles. After eclosion, adult mosquitoes were maintained in ventilated cages at 28 °C and 75% relative humidity (RH) under 12 h:12 h light/dark cycles. These mosquitoes were only fed with 8% glucose solution.

### Single-cell preparation from midguts

For each experimental replicate, 40 midguts were dissected from female mosquitoes at 7 days after eclosion, then washed and temporarily stored in Schneider’s Drosophila Medium (Gibco, USA) on ice. After dissection, the medium was discarded, and the midguts were digested in 400 μl of dissociation buffer (1 mg/ml collagenase/dispase in 1 × PBS) in a shaking incubator for 20–30 min (mixed samples every five minutes) at room temperature. Then, 400 μl of Schneider’s Drosophila Medium was added to the microtube and mixed well. The resulting 800 μl of the cell suspension was filtered with a 40 μm Flowmi Cell Strainer (SP Scienceware, USA) to remove larger cell debris. To remove impurities and smaller cell debris, the filter was centrifuged over a 1.12 g/ml density solution made with OptiPrep (Serumwerk Bernburg, German) at 400 × g, 4 °C for 20 min. A viable cell band was collected and subjected to an additional centrifugation under the same conditions. The cell precipitation was washed with 1 × PBS for three times and then resuspended in 200 μl of Schneider’s Drosophila Medium. For quality control (QC), cell concentration, viability and aggregation rate were measured by staining with Calcein/PI Cell Viability/Cytotoxicity Assay Kit (Beyotime, China) and single cells were counted with a hemocytometer under a fluorescence microscope. Cell suspension samples with viability ≥80% and aggregation rate <5% were used for subsequent scRNA-seq.

### Library construction and sequencing

According to the manufacturer’s guidelines, the single cell concentration was adjusted to 1000–1400 cells/μl prior to loading onto a Chromium microfluidic chip (10 × Genomics). The cDNA synthesis of captured cells and sequencing library construction were performed with Single Cell 3’ v3 Chemistry. The constructed libraries were sequenced with NovaSeq 6000 system in accordance with the manufacturer’s instructions.

### Single-cell RNA sequencing analysis

**Fig. 1:**
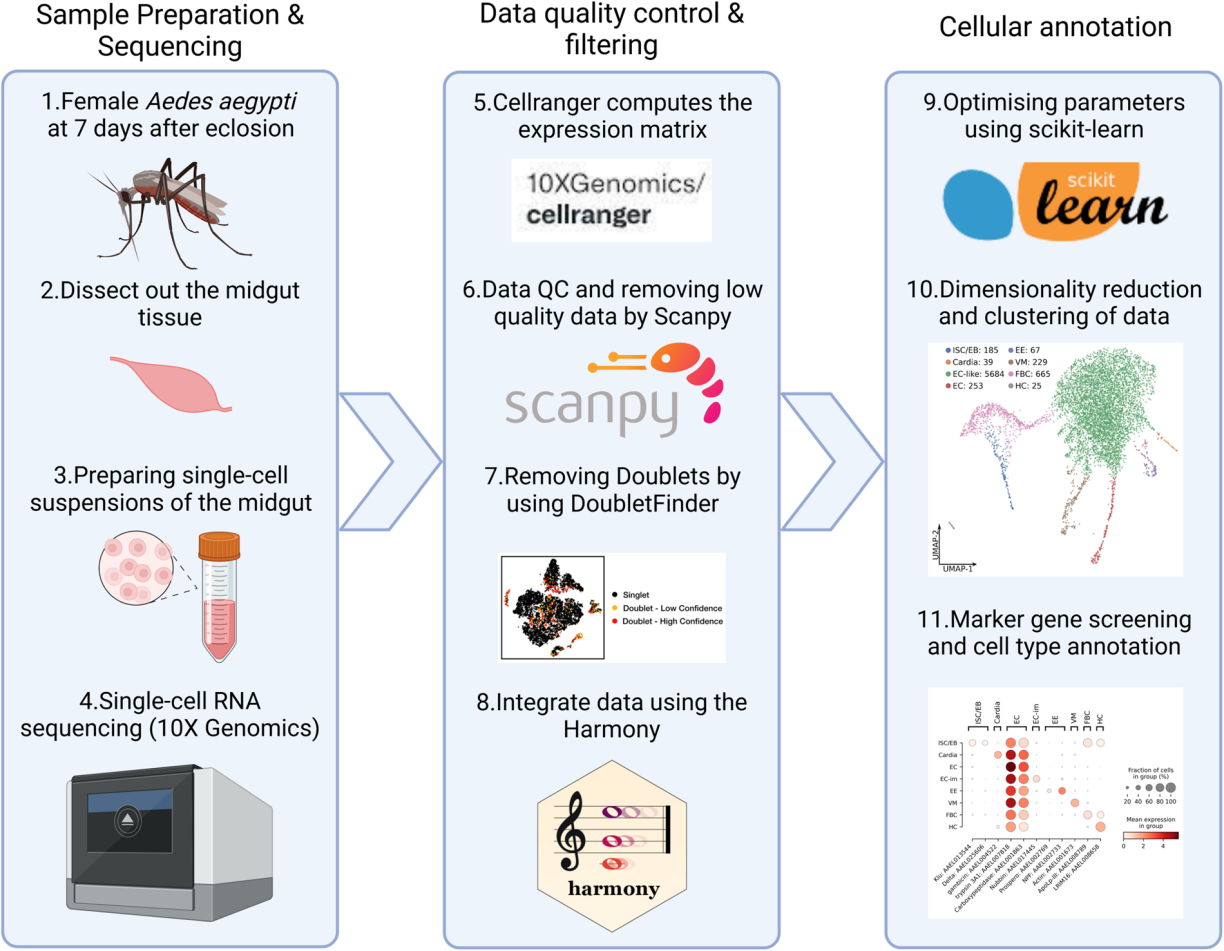
Flow diagram of the scRNA-seq and data analysis.

For each replicate, expression matrix calculation, data filtering, normalization, dimensionality reduction by PCA (principal component analysis), UMAP clustering, and doublet screening were performed separately. The valid cell barcodes, unique molecular identifiers (UMI) and gene features were checked, raw reads were then qualified, demultiplexed and mapped to the genome (Genome assembly AaegL5.3) of *Ae. aegypti* from VectorBase (https://vectorbase.org/common/downloads/release-65/AaegyptiLVP_AGWG/) using Cell Ranger (v7.2.0)^23^. Then, the feature expression matrix was constructed by Cell Ranger, and scRNA-seq analysis was performed with Scanpy (v1.9.5)^24^. The low-quality cells with unique gene numbers >2500 or <100 and a proportion of mitochondrial transcripts >30% were filtered out^13^. The filtered data were log normalized and the top 2000 highly variable genes were used for PCA dimension reduction. The silhouette_score function of scikit-learn (v1.3.0)^25^ was used to calculate the silhouette coefficients of the clustering results for different numbers of principal components and resolutions to select the optimal parameters for clustering. The optimal parameters of rep1 (first 21 principal components, resolution = 0.1) and rep2 (first 14 principal components, resolution = 0.1) were used for clustering, respectively. DoubletFinder (v2.0.3)^26^ was used to identify doublets. After filtering out the doublet data, rep1 and rep2 were integrated as merged data using Harmony (v0.0.9)^27^. The merged data was subjected to data scaling, PCA, and clustering using the optimal parameters (first 18 principal components, resolution = 0.1). The filter_rank_genes_groups function in Scanpy was used to calculate the specifically expressed genes for each cell cluster. Cell types were annotated by referring to the published single-cell (nucleus) RNA-seq studies of *Ae. aegypti*^13,15^. Data visualization was performed by Scanpy, Matplotlib (v3.8.0)^28^ and Seaborn (v0.13.0)^29^ (Fig. 1).

## Data Records

The sequencing raw data and scRNA-seq data are available on Gene Expression Omnibus (GEO). GSE accession: GSE246612 with sample GSM7872696 and GSM7872697 for rep1 and rep2^30^. The GEO samples contain the files as follows:

**raw sequencing data files:**

*SRR26591148* (for rep1)*, SRR26591149* (for rep2): the raw sequencing files with FASTQ format available in SRA run selector

**10x Genomics scRNA-seq data files:**

*GSM7872696_sub_rep1_barcodes.tsv.gz, GSM7872697_sub_rep2_barcodes.tsv.gz*: cell barcodes identified by the downstream analysis

*GSM7872696_sub_rep1_features.tsv.gz, GSM7872697_sub_rep2_features.tsv.gz*: gene features identified by the downstream analysis

*GSM7872696_sub_rep1_matrix.mtx.gz, GSM7872697_sub_rep2_matrix.mtx.gz*: a matrix that describes gene expression in each cell

## Technical Validation

**Fig. 2:**
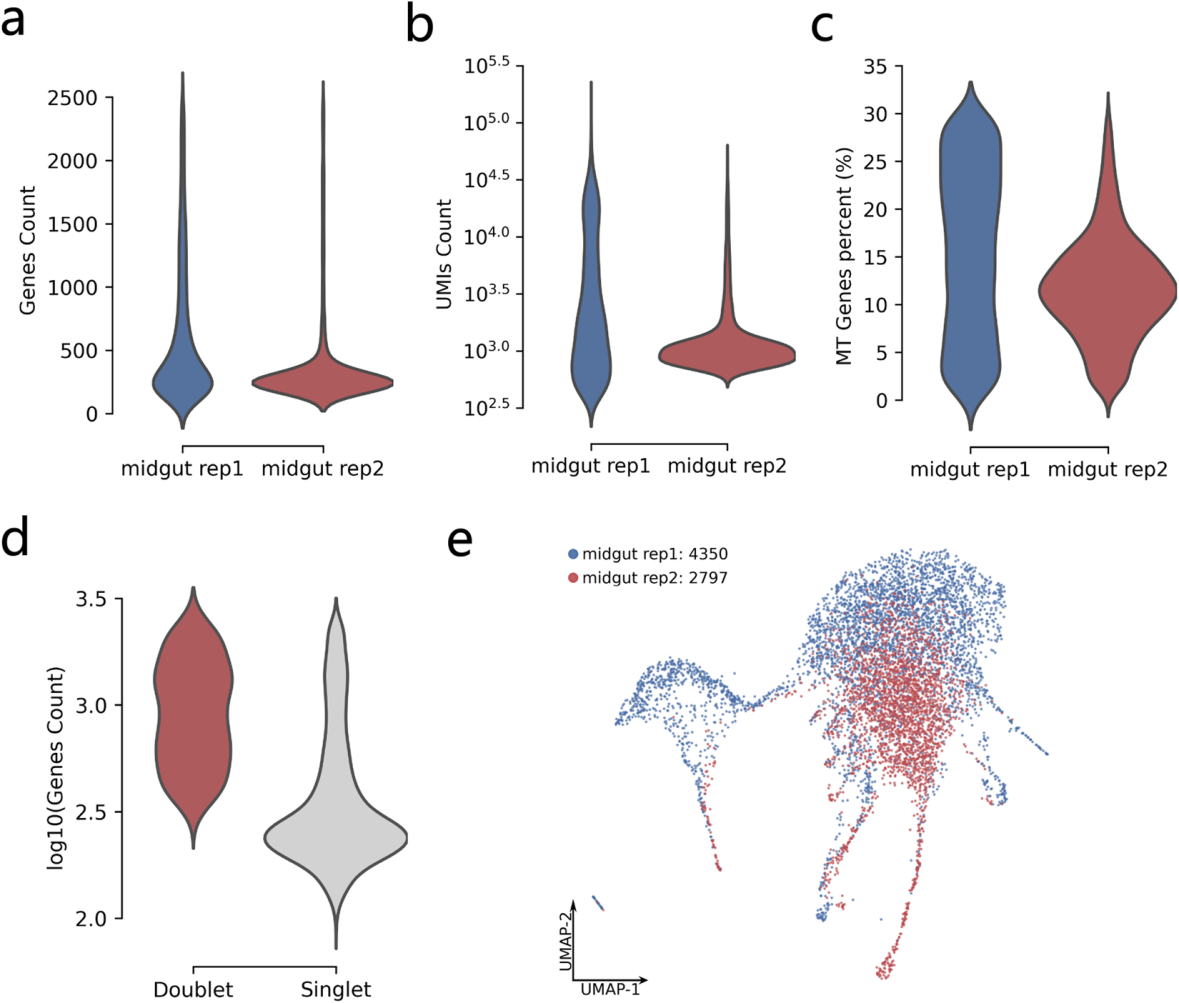
Summary of scRNA-seq data of *Ae. aegypti* midgut after filtering low-quality cells. (**a**) Distribution of the number of genes in each replicate after data filtering. (**b**) Distribution of the number of UMIs in each replicate after data filtering. (**c**) Distribution of mitochondrial gene percentage in each replicate after data filtering. (**d**) Distribution of gene counts in doublets and singlets. (**e**) Distribution of individual replicates in UMAP. The violin plot shows the range of distribution of single-cell QC data values. The widest area (bulb shape) of the violin plot shows the range of the main distribution of QC data values.

Since the transcriptomes of mosquito cells are different from those of mammals, we established single-cell QC standards for mosquito transcriptomes by referring to a scRNA-seq study of hemocytes of *An. gambiae* and *Ae. aegypti*^13^. At the stage of calculating the expression matrix, we used the–force-cells parameter of Cell Ranger for rep2 and set the value of forced cells at 3064 to ensure as many cells detected as possible while maintaining the cell quality (median value of genes around 270). Before QC, 383,640,091 and 270,662,393 reads were obtained in the raw data for rep1 and rep2, respectively (Table 1). The mean reads per cell for rep1 and rep2 are 31,840 and 88,336, respectively, with median genes per cell of 212 and 263, respectively (Table 2). After QC and filtering of the raw data, the median genes per cell in rep1 and rep2 are 359 and 251 (Fig. 2a), and the median numbers of UMIs of cells are 1,637 and 1,009, respectively (Fig. 2b). The percentage of mitochondrial genes in both replicates was less than 30% (Fig. 2c). Detection of doublets from the data showed that the number of genes in doublet samples was greater than in singlet samples (Fig. 2d), which was characteristic of doublet samples. In total, 298 doublets and 7,147 singlets were identified. Among these singlets, 4,350 cells belonged to rep1 and 2,797 to rep2. The UMAP results showed that most of the cells in rep2 were overlapped with cells in rep1, some cells in rep1 were not overlapped with cells in rep2 because there are much more cells from rep1 (Fig. 2e).

**Table 1:** Sequencing summary of raw scRNA-seq data.

Item	Midgut rep1	Midgut rep2
Reads	383,640,091	270,662,393
Q30 Bases in Barcode	96.20%	91.40%
Q30 Bases in RNA Read	90.00%	92.10%
Q30 Bases in UMI	95.60%	90.80%
Confident Mapping to Genome	81.70%	55.90%
Confident Mapping to Transcriptome	79.60%	53.50%

**Table 2:** Quality summary of cell-based scRNA-seq data before filtering low-quality cells.

Item	Midgut rep1	Midgut rep2
Estimated number of cells	12,049	3,064
Mean reads per cell	31,840	88,336
Median UMI counts per cell	1,012	1,154
Median genes per cell	212	263
Total genes detected	13,385	12,449
Mitochondria ratio	35.44%	11.43%

**Fig. 3:**
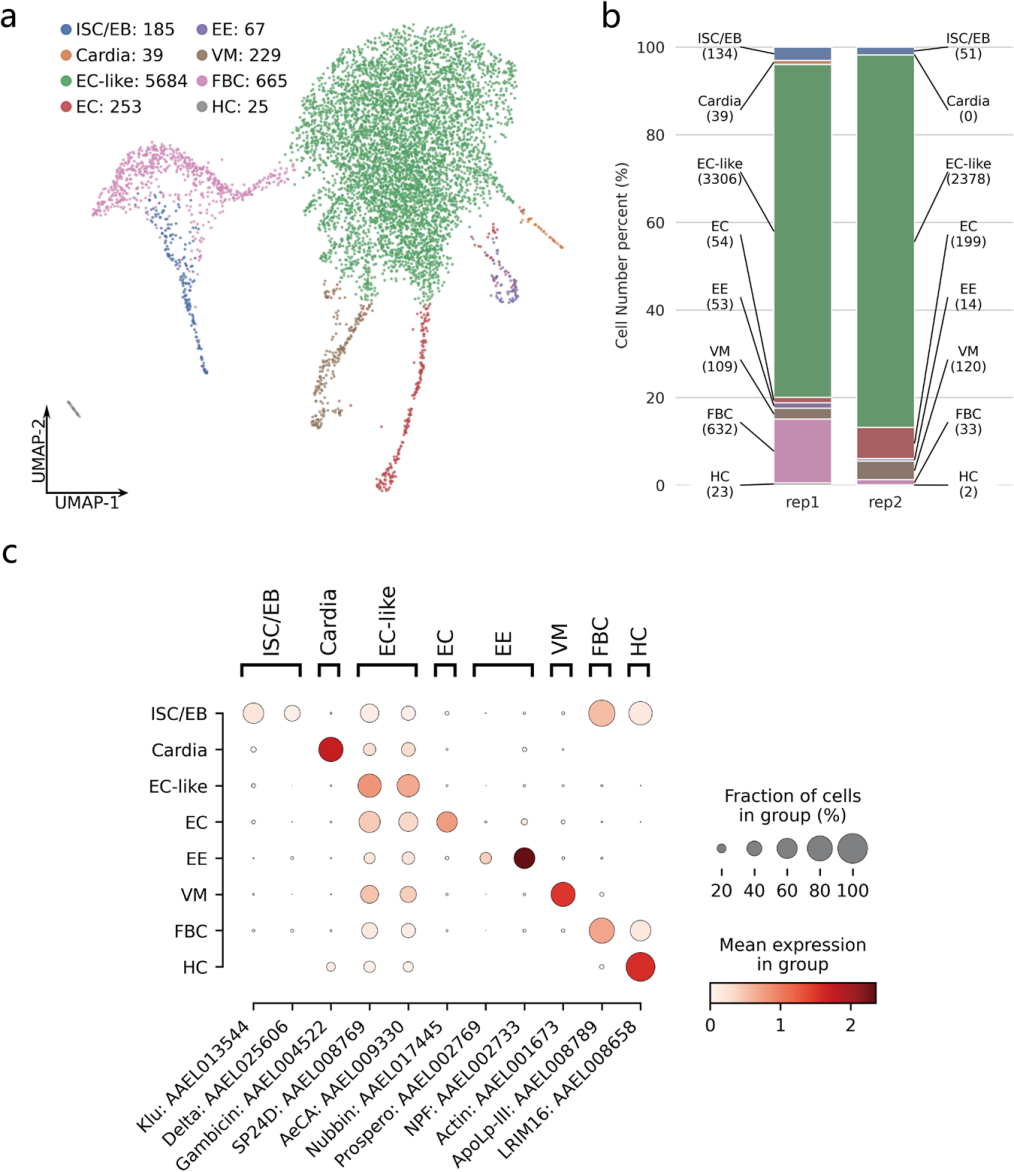
Cell type annotation for cell clusters. (**a**) Cell type annotation results. The number of cells is labeled after the cluster name. (**b**) Percentage of cells with replicate 1 and replicate 2 in each cell type. The number of cells is labeled in parentheses. (**c**) Expression of cell type marker genes in each cell cluster. Intestinal stem cells/enteroblasts (ISC/EB), cardia cells (Cardia), enterocytes (EC), enterocytes-like (EC-like), enteroendocrine cells (EE), visceral muscle (VM), fat body cells (FBC) and hemocytes (HC).

**Fig. 4:**
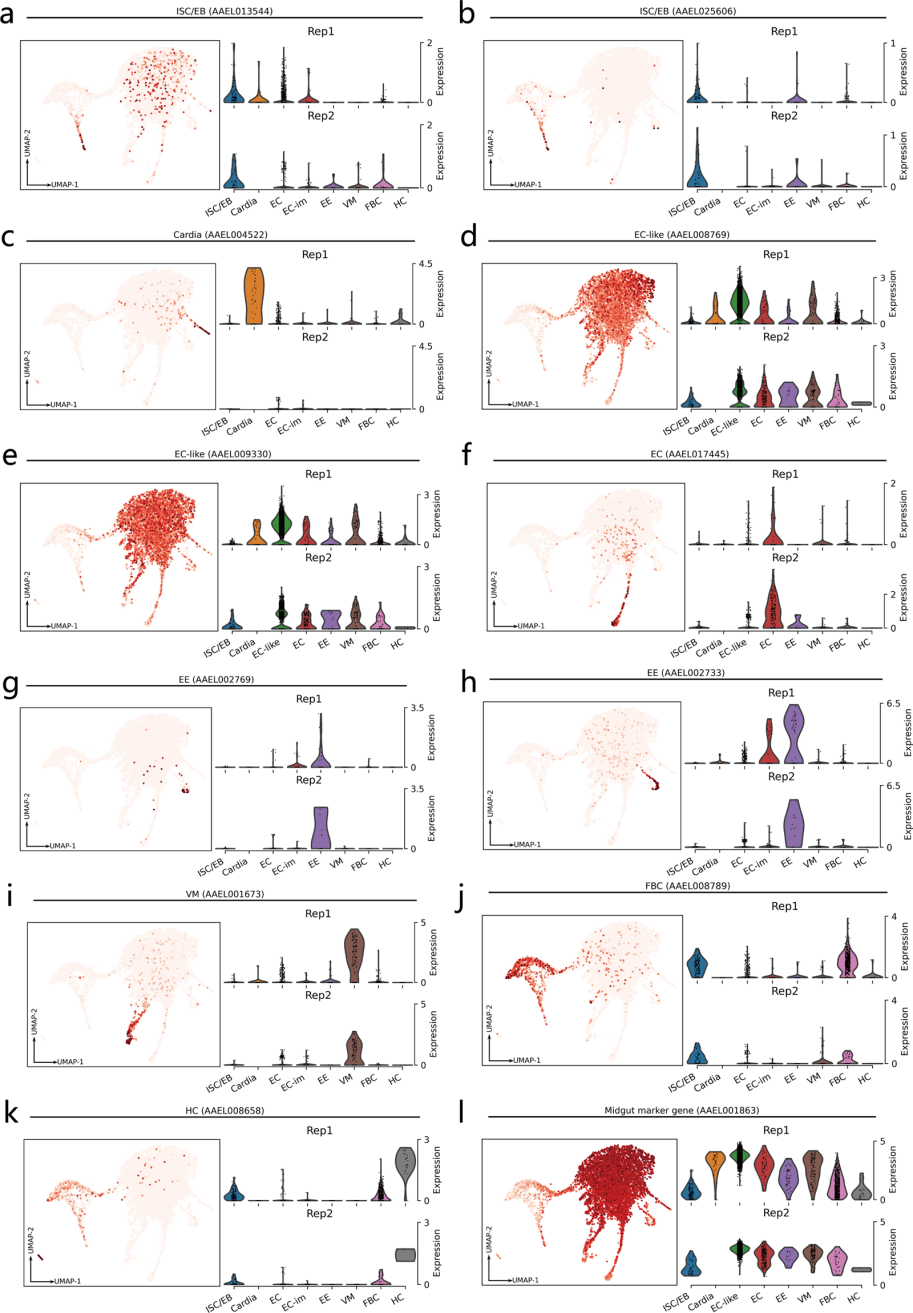
Marker gene analysis by cell type. (**a**–**l**) Each subplot corresponds to a marker gene. The expression of marker genes in all cells and their cell specificity in each replicate are shown in the subplots. Dark and light red in the dot plots indicate highly and lowly expressed genes, respectively.

We successfully annotated 8 cell types, ISC/EB, Cardia, EC-like, EC, EE, VM, FBC, and HC (Fig. 3a, Table 3, Table S1). EC-like cell cluster is the biggest one in both replicates (Fig. 3b). ISC/EB, EE, FBC, and HC contain a greater proportion of cells in rep1, while EC contains a greater proportion of cells in rep2 (Fig. 3b). Cardia was only detected in rep1 (Fig. 3b). These results indicate that the two replicates were mostly consistent in terms of cell type. We identified marker genes for each cell type^13,15^, which can be used to clearly distinguish each cell type (Fig. 3c). To confirm the cell specificity of these marker genes in each replicate, we calculated the expression of marker genes between the replicates (Fig. 4a-k). The results showed that the expression of most of the marker genes for rep2 was lower than that for rep1, but the expression trends across cell types were consistent across replicates. As EC-like cells contain the highest number of cells in both replicates, it is necessary to further clarify its midgut cell characteristics. We compared the expression of the midgut marker gene carboxypeptidase (AAEL001863)^31^ among cell types (Fig. 4l). The results showed that the expression of carboxypeptidase was much higher in EC-like cells than in other cell types, suggesting that most of the detected cells have distinct midgut cell characteristics. These results indicate that most of the cell types identified in the two replicates are consistent, and the vast majority of the cells have distinct midgut cell characteristics.

**Table 3:** Cell type marker genes reported in previous studies. The information for marker gene description was obtained from the NCBI database.

Cell type	Marker gene accession	Marker gene name	Marker gene description	Marker in *Drosophila*
ISC/EB	AAEL013544, AAEL025606	Klu^15^, Delta^15^	Chorion transcription factor Cf2, Neurogenic locus protein delta	Yes, Yes
Cardia	AAEL004522	Gambicin^15^	Uncharacterized LOC5564993	No
EC-like	AAEL008769, AAEL009330	SP24D^15^, AeCA^15^	Serine protease SP24D, Carbonic anhydrase	No, No
EC	AAEL017445	Nubbin^15^	Protein nubbin	Yes
EE	AAEL002769, AAEL002733	Prospero^15^, NPF^15^	Homeobox protein prospero, Neuropeptide F	No, Yes
VM	AAEL001673	Actin^15^	Muscle actin	Yes
FBC	AAEL008789	ApoLp-III^13^	Apolipophorin-3	No
HC	AAEL008658	LRIM16 (cDIP)^13^	Common Dpr-interacting protein	No

## Usage Notes

Variabilities in the experimental replicates were observed in both previous scRNA-seq studies on insects and this study^14,32^. The variances may be influenced by factors such as mosquitoes from different batches, different dissection techniques performed by different persons and the time for storing midguts on ice before dissociation. The mosquito midgut scRNA-seq data in this study were uploaded to the GEO database, which includes the raw FASTQ files and the expression matrix files processed by Cell Ranger software. Our scripts have been uploaded to Github (see Code Availability section for details) to allow other researchers to reproduce the results or combine them with other data for further analysis.

### Supplementary information

Supplementary Table S1

## Data Availability

The scRNA-seq analysis scripts for this study have been uploaded to GitHub (https://github.com/yingHH/Aaedes_midgut_scRNA-Seq).
